# l-Valine ethyl ester hydro­chloride

**DOI:** 10.1107/S2414314625010478

**Published:** 2025-11-28

**Authors:** Erin Van Rooyen, Eric Cyriel Hosten, Richard Betz

**Affiliations:** a Nelson Mandela University, Summerstrand Campus, Department of Chemistry, University Way, Summerstrand, PO Box 77000, Port Elizabeth, 6031, South Africa; University of Aberdeen, United Kingdom

**Keywords:** crystal structure, amino acid, protecting group

## Abstract

The title compound [systematic name: (2*S*)-1-ethoxy-3-methyl-1-oxobutan-2-aminium chloride], C_7_H_16_NO_2_^+^·Cl^−^, is the hydro­chloride salt of the ethyl ester of l-valine. In the crystal, classical hydrogen bonds of the N—H⋯Cl type are observed alongside C—H⋯O and C—H⋯Cl contacts, connecting the entities of the asymmetric unit into sheets lying perpendicular to the crystallographic *a* axis.

## Abstract 2

The title compound is the hydro­chloride salt of the ethyl ester of l-valine. Inter­molecular inter­actions connect the entities of the asymmetric unit into sheets lying parallel the the *bc* plane.

## Graphical abstract

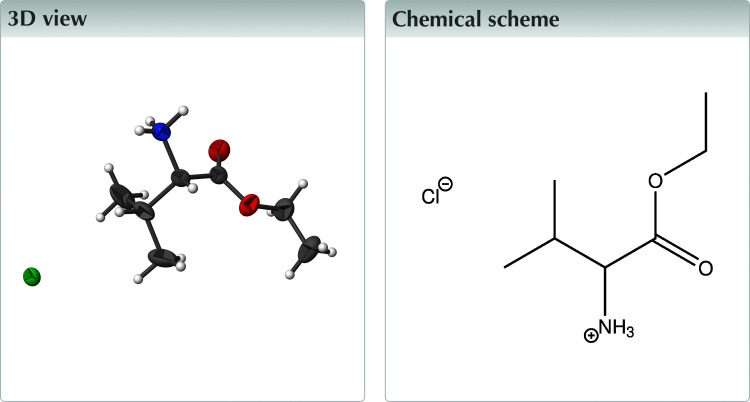

## Structure description

Amino acids play a pivotal role in the human metabolism and represent a crucial macronutrient class at the centre of all higher life. Natural representatives of this compound class appear as the l-configured stereosiomers in organisms and give rise to the chiral nature of proteins (McMurry, 2008 ▸). As a result of their bidentate nature, they have found ample use as chelating ligands in transition-metal chemistry whose denticity can be fine-tuned by varying the pH of the reaction mixture under investigation (Gade, 1998 ▸). Potential donor sites on certain amino acid side chains can further diversify the bonding behaviour and give rise to unique bonding patterns. One way to simplify the variety of bonding modes to be encountered is to block the acid group by means of esterification (Becker *et al.*, 2000 ▸). At the onset of a study around the coordination and condensation behaviour of amino acids and certain derivatives thereof towards selected transition metals and main group elements, the metrical parameters of starting materials need to be established to allow for comparative studies regarding the influence of binding on bond lengths and angles. Structural information for the ethyl ester hydro­chlorides of phenyl­glycine (Brunner *et al.*, 2021 ▸), nitroso cysteine (Yi *et al.*, 2005 ▸, 2016 ▸), glycine (He *et al.*, 2010 ▸), cysteine (Haas, 1965 ▸; Gorbitz, 1989 ▸; Defonsi Lestard *et al.*, 2013 ▸) is apparent in the literature. Furthermore, we have elucidated the mol­ecular and crystal structures of the methyl ester hydro­chloride salt of l-valine (Betz *et al.*, 2011 ▸), metacholine chloride (Muller *et al.*, 2021 ▸), as well as the hydro­chloride salt of benzyl­glycine (Hosten *et al.*, 2011 ▸). As an extension of these studies, we now report the structure of the title compound, C_7_H_16_NO_2_^+^·Cl^−^ (**I**).

**Figure 1:**
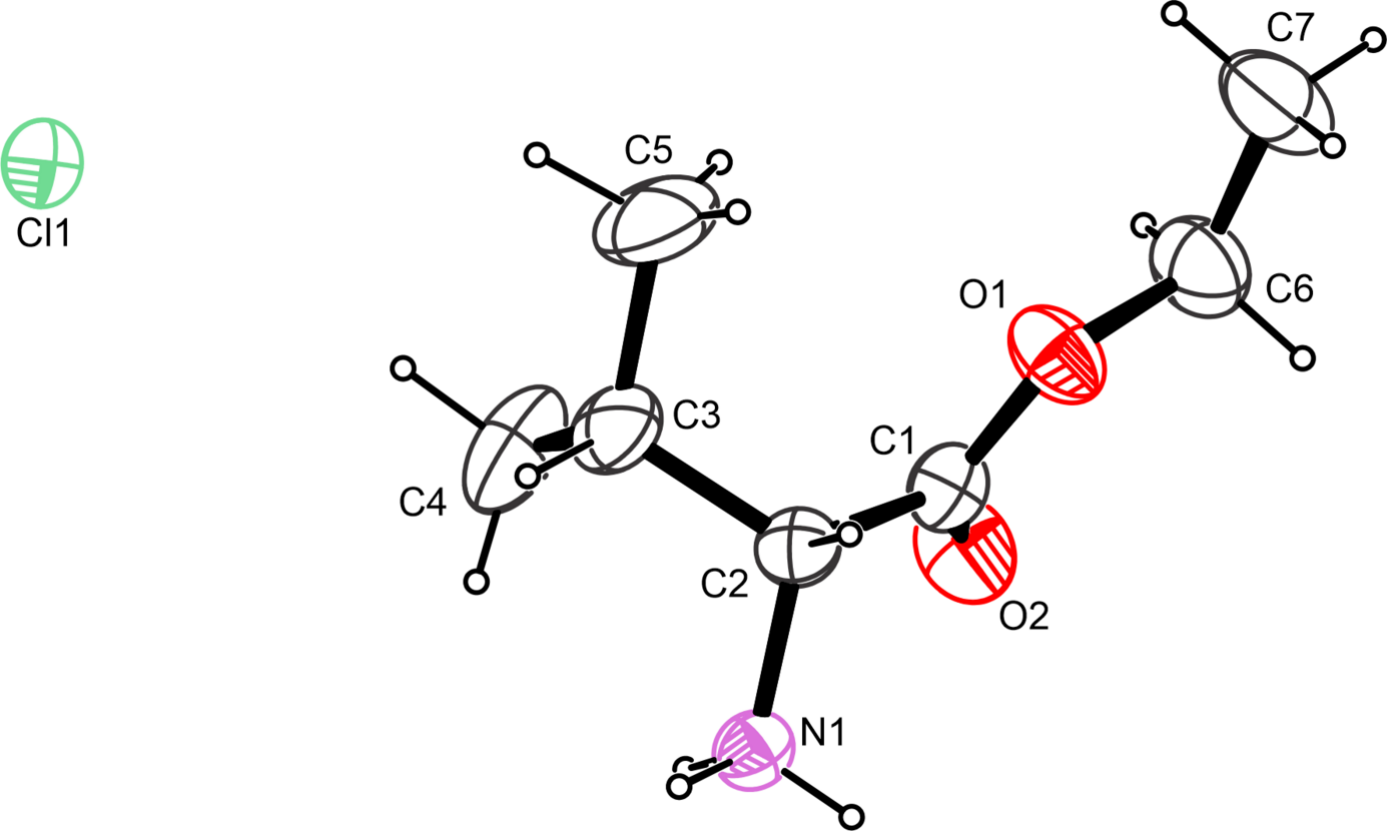
The mol­ecular structure of (**I**) with displacement ellipsoids drawn at the 50% probability level.

The structure solution shows a derivative of l-valine with the carboxyl group converted into the ethyl ester (Fig. 1 ▸). The absolute structure in space group *P*2_1_ is well established and, as expected, the stereogenic atom C2 has an *S* configuration. The amino group shows protonation whose positive charge has been counterbalanced by a chloride anion. The ethyl side chain, the carboxyl group as well as the nitro­gen-bearing carbon atom are close to co-planar with the largest deviation from the least-squares plane as defined by the non-hydrogen atoms of the aforementioned moieties is 0.104 (2) Å for the ethereal oxygen atom. Selected torsion angles include O1—C1—C2—N1 = 148.9 (2)°, C1—C2—C3—C4 = −64.3 (4)° and C1—O1—C6—C7 = −170.3 (3)°. Otherwise, all bond lengths and angles are found to be in good agreement with values for comparable compounds whose metrical parameters have been deposited with the Cambridge Structural Database (Groom *et al.*, 2016 ▸).

**Figure 2:**
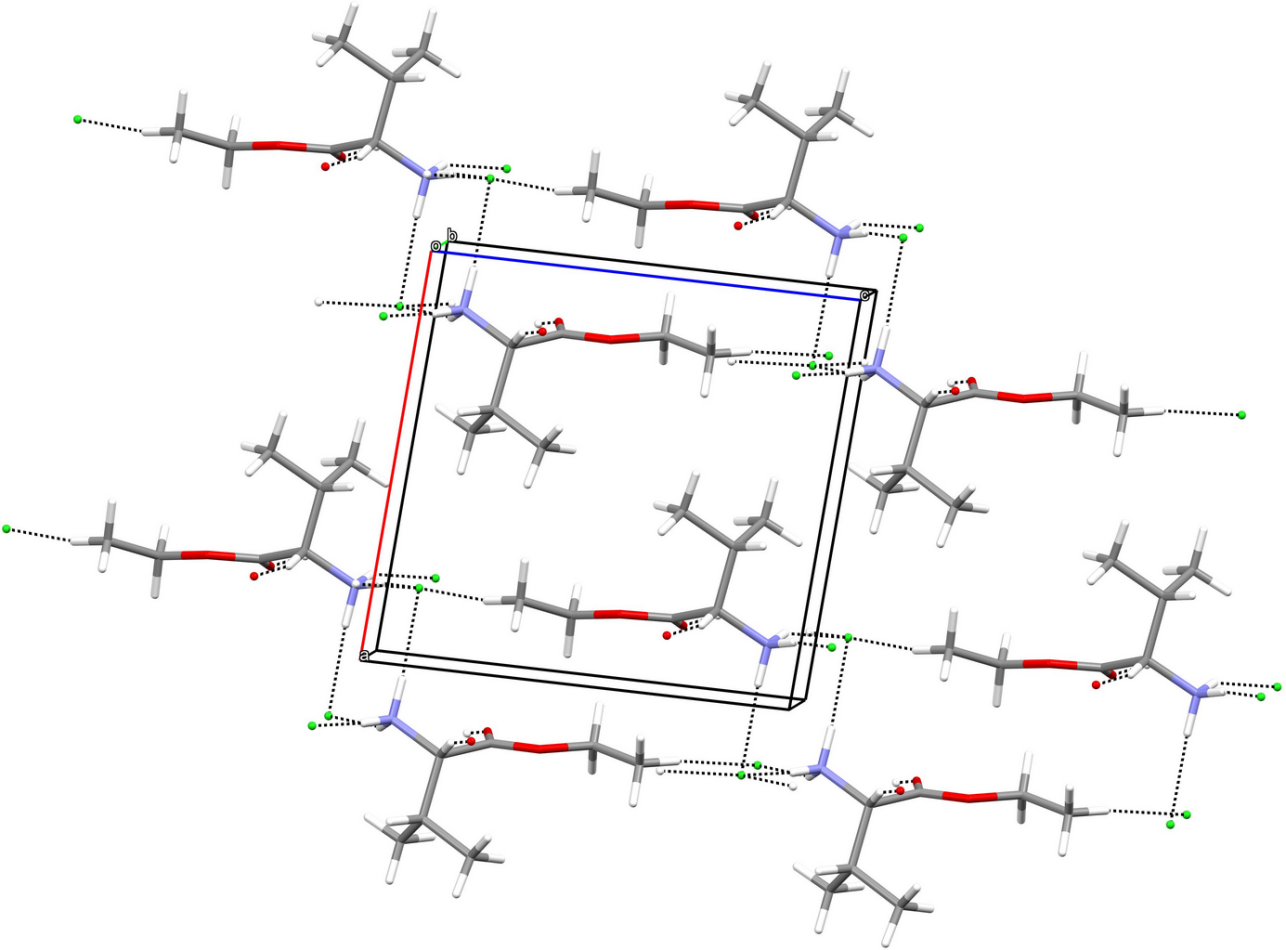
Inter­molecular contacts (black dashed lines) in (**I**), viewed approximately along [010].

In the crystal of (**I**), classical hydrogen bonds of the N—H⋯Cl type are observed as well as C—H⋯O and C—H⋯Cl contacts whose range falls by more than 0.1 Å below the sum of van der Waals radii of the atoms participating in them (Table 1 ▸). These are established by all the nitro­gen-bound hydrogen atoms as donors in the case of the classical hydrogen bonds (which generate [010] chains), as well as the hydrogen atom of the stereocentre and the ketonic oxygen atom in case of the C—H⋯O contacts. The chlorine-supported C—H inter­actions stem from one of the hydrogen atoms of the methyl group in the ester side chain. In terms of graph-set analysis (Etter *et al.*, 1990 ▸; Bernstein *et al.*, 1995 ▸), the descriptor for these inter­actions is *DDDDC*^1^_1_(4) on the unary level. In total, the entities of the asymmetric unit are connected to sheets lying perpendicular to the crystallographic *a* axis with the chloride anions forming channels along the crystallographic *b-*axis direction (Fig. 2 ▸).

## Synthesis and crystallization

The compound was obtained commercially (Fluka). Crystals suitable for the diffraction study were obtained upon prolonged storage of the compound at room temperature in a tightly sealed glass bottle.

## Refinement

Crystal data, data collection and structure refinement details are summarized in Table 2 ▸.

## Supplementary Material

Crystal structure: contains datablock(s) I. DOI: 10.1107/S2414314625010478/hb4545sup1.cif

Structure factors: contains datablock(s) I. DOI: 10.1107/S2414314625010478/hb4545Isup2.hkl

CCDC reference: 2505000

Additional supporting information:  crystallographic information; 3D view; checkCIF report

## Figures and Tables

**Table 1:** Hydrogen-bond geometry (Å, °)

*D*—H⋯*A*	*D*—H	H⋯*A*	*D*⋯*A*	*D*—H⋯*A*
N1—H11⋯Cl1^i^	0.91 (3)	2.34 (4)	3.192 (3)	157 (3)
N1—H12⋯Cl1^ii^	0.81 (4)	2.38 (4)	3.133 (3)	155 (3)
N1—H13⋯Cl1^iii^	0.94 (3)	2.19 (3)	3.122 (2)	175 (3)
C2—H2⋯O2^iv^	1.00	2.40	3.377 (3)	165
C7—H7*B*⋯Cl1^v^	0.98	2.82	3.760 (4)	160

Symmetry codes: (i) 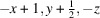; (ii) 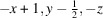; (iii) 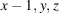; (iv) 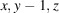; (v) 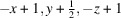.

**Table 2:** Experimental details

Crystal data	
Chemical formula	C_7_H_16_NO_2_^+^·Cl^−^
*M*_r_	181.66
Crystal system, space group	Monoclinic, *P*2_1_
Temperature (K)	200
*a*, *b*, *c* (Å)	9.7222 (7), 5.3577 (4), 10.1105 (8)
β (°)	93.499 (3)
*V* (Å^3^)	525.66 (7)
*Z*	2
Radiation type	Mo *K*α
μ (mm^−1^)	0.33
Crystal size (mm)	0.24 × 0.20 × 0.16

Data collection	
Diffractometer	Bruker D8 Quest CCD, software *APEX5*
Absorption correction	Multi-scan (*SADABS*; Krause *et al.*, 2015 ▸)
*T*_min_, *T*_max_	0.681, 0.746
No. of measured, independent and observed [*I* > 2σ(*I*)] reflections	30362, 2592, 2378
*R*_int_	0.050
(sin θ/λ)_max_ (Å^−1^)	0.666

Refinement	
*R*[*F*^2^ > 2σ(*F*^2^)], *wR*(*F*^2^), *S*	0.036, 0.094, 1.11
No. of reflections	2592
No. of parameters	115
No. of restraints	1
H-atom treatment	H atoms treated by a mixture of independent and constrained refinement
Δρ_max_, Δρ_min_ (e Å^−3^)	0.39, −0.19
Absolute structure	Flack *x* determined using 983 quotients [(*I*^+^)−(*I*^−^)]/[(*I*^+^)+(*I*^−^)] (Parsons *et al.*, 2013 ▸)
Absolute structure parameter	0.037 (17)

Computer programs: *APEX2* and *SAINT* (Bruker, 2014 ▸), *SHELXS7* (Sheldrick 2008 ▸), *ORTEP-3 for Windows* (Farrugia, 2012 ▸), *Mercury* (Macrae *et al.*, 2020 ▸), *SHELXL2019/3* (Sheldrick, 2015 ▸) and *PLATON* (Spek, 2020 ▸).

## full crystallographic data

### (2S)-1-Ethoxy-3-methyl-1-oxobutan-2-aminium chloride Crystal data

**Table:**

C_7_H_16_NO_2_^+^·Cl^−^	*F*(000) = 196
*M**_r_* = 181.66	*D*_x_ = 1.148 Mg m^−^^3^
Monoclinic, *P*2_1_	Mo *K*α radiation, λ = 0.71073 Å
*a* = 9.7222 (7) Å	Cell parameters from 9975 reflections
*b* = 5.3577 (4) Å	θ = 3.0–28.3°
*c* = 10.1105 (8) Å	µ = 0.33 mm^−^^1^
β = 93.499 (3)°	*T* = 200 K
*V* = 525.66 (7) Å^3^	Block, colourless
*Z* = 2	0.24 × 0.20 × 0.16 mm

### (2S)-1-Ethoxy-3-methyl-1-oxobutan-2-aminium chloride Data collection

**Table:**

Bruker D8 Quest CCD, software APEX5 diffractometer	2378 reflections with *I* > 2σ(*I*)
φ and ω scans	*R*_int_ = 0.050
Absorption correction: multi-scan (SADABS; Krause *et al.*, 2015)	θ_max_ = 28.3°, θ_min_ = 2.0°
*T*_min_ = 0.681, *T*_max_ = 0.746	*h* = −12→12
30362 measured reflections	*k* = −7→7
2592 independent reflections	*l* = −13→13

### (2S)-1-Ethoxy-3-methyl-1-oxobutan-2-aminium chloride Refinement

**Table:**

Refinement on *F*^2^	Secondary atom site location: difference Fourier map
Least-squares matrix: full	Hydrogen site location: mixed
*R*[*F*^2^ > 2σ(*F*^2^)] = 0.036	H atoms treated by a mixture of independent and constrained refinement
*wR*(*F*^2^) = 0.094	*w* = 1/[σ^2^(*F*_o_^2^) + (0.0483*P*)^2^ + 0.1046*P*] where *P* = (*F*_o_^2^ + 2*F*_c_^2^)/3
*S* = 1.11	(Δ/σ)_max_ = 0.001
2592 reflections	Δρ_max_ = 0.39 e Å^−^^3^
115 parameters	Δρ_min_ = −0.19 e Å^−^^3^
1 restraint	Absolute structure: Flack *x* determined using 983 quotients [(*I*^+^)-(*I*^-^)]/[(*I*^+^)+(*I*^-^)] (Parsons *et al.*, 2013)
Primary atom site location: structure-invariant direct methods	Absolute structure parameter: 0.037 (17)

### (2S)-1-Ethoxy-3-methyl-1-oxobutan-2-aminium chloride Special details

**Table:**

Geometry. All esds (except the esd in the dihedral angle between two l.s. planes) are estimated using the full covariance matrix. The cell esds are taken into account individually in the estimation of esds in distances, angles and torsion angles; correlations between esds in cell parameters are only used when they are defined by crystal symmetry. An approximate (isotropic) treatment of cell esds is used for estimating esds involving l.s. planes.
Refinement. The carbon-bound H atoms were placed in calculated positions (C—H = 0.99 Å for the methylene group, C—H = 1.00 Å for the methine groups) and were included in the refinement in the riding model approximation, with *U*_iso_(H) = 1.2*U*_eq_(C). The H atoms of the methyl groups were allowed to rotate with a fixed angle around the C—C bond to best fit the experimental electron density (HFIX 137 in the *SHELX* program suite (Sheldrick, 2015), with *U*_iso_(H) = 1.5*U*_eq_(C). All three nitrogen-bound H atoms were located in a difference Fourier map and refined freely.

### (2S)-1-Ethoxy-3-methyl-1-oxobutan-2-aminium chloride Fractional atomic coordinates and isotropic or equivalent isotropic displacement parameters (Å^2^)

**Table:**

	*x*	*y*	*z*	*U*_iso_*/*U*_eq_	
O1	0.1799 (2)	0.5810 (6)	0.42612 (16)	0.0430 (4)	
O2	0.1606 (2)	0.9078 (4)	0.2891 (2)	0.0476 (5)	
N1	0.14778 (19)	0.5828 (6)	0.07661 (19)	0.0315 (4)	
H11	0.175 (3)	0.734 (6)	0.048 (3)	0.028 (8)*	
H12	0.163 (3)	0.488 (7)	0.017 (4)	0.041 (10)*	
H13	0.052 (3)	0.590 (8)	0.084 (3)	0.042 (7)*	
C1	0.1815 (2)	0.6913 (5)	0.3087 (3)	0.0323 (5)	
C2	0.2186 (2)	0.5065 (5)	0.2043 (3)	0.0315 (5)	
H2	0.182912	0.338817	0.228863	0.038*	
C3	0.3750 (3)	0.4843 (6)	0.1889 (3)	0.0446 (7)	
H3	0.388485	0.353065	0.120594	0.053*	
C4	0.4394 (3)	0.7226 (8)	0.1406 (5)	0.0696 (11)	
H4A	0.426621	0.856780	0.204623	0.104*	
H4B	0.395174	0.769270	0.054479	0.104*	
H4C	0.538125	0.695948	0.131565	0.104*	
C5	0.4487 (4)	0.3945 (9)	0.3184 (4)	0.0709 (11)	
H5A	0.542475	0.341653	0.301441	0.106*	
H5B	0.398092	0.253446	0.353508	0.106*	
H5C	0.452595	0.530938	0.383063	0.106*	
C6	0.1598 (4)	0.7340 (7)	0.5419 (3)	0.0513 (8)	
H6A	0.064320	0.799024	0.538592	0.062*	
H6B	0.224035	0.877473	0.544488	0.062*	
C7	0.1861 (4)	0.5768 (10)	0.6606 (3)	0.0615 (9)	
H7A	0.121992	0.435409	0.657131	0.092*	
H7B	0.172734	0.676039	0.740249	0.092*	
H7C	0.280990	0.514478	0.663367	0.092*	
Cl1	0.82750 (5)	0.58922 (7)	0.08644 (5)	0.03471 (17)	

### (2S)-1-Ethoxy-3-methyl-1-oxobutan-2-aminium chloride Atomic displacement parameters (Å^2^)

**Table:**

	*U*^11^	*U*^22^	*U*^33^	*U*^12^	*U*^13^	*U*^23^
O1	0.0631 (11)	0.0339 (9)	0.0324 (8)	0.0021 (11)	0.0057 (7)	−0.0014 (10)
O2	0.0681 (14)	0.0269 (10)	0.0487 (11)	0.0080 (9)	0.0104 (10)	0.0010 (9)
N1	0.0311 (9)	0.0284 (10)	0.0351 (9)	0.0023 (12)	0.0015 (7)	−0.0016 (14)
C1	0.0301 (12)	0.0309 (12)	0.0361 (13)	−0.0009 (10)	0.0032 (9)	0.0002 (10)
C2	0.0329 (12)	0.0247 (11)	0.0368 (13)	0.0029 (9)	0.0013 (9)	0.0004 (9)
C3	0.0333 (13)	0.0491 (16)	0.0509 (16)	0.0153 (12)	−0.0007 (11)	−0.0099 (13)
C4	0.0291 (15)	0.074 (3)	0.107 (3)	0.0004 (16)	0.0145 (17)	0.009 (2)
C5	0.058 (2)	0.083 (3)	0.069 (2)	0.030 (2)	−0.0199 (17)	−0.005 (2)
C6	0.0631 (19)	0.054 (2)	0.0368 (15)	0.0060 (15)	0.0050 (13)	−0.0086 (14)
C7	0.080 (2)	0.072 (2)	0.0323 (13)	−0.018 (3)	0.0029 (13)	−0.001 (2)
Cl1	0.0342 (3)	0.0331 (3)	0.0373 (3)	0.0006 (3)	0.00536 (19)	−0.0008 (3)

### (2S)-1-Ethoxy-3-methyl-1-oxobutan-2-aminium chloride Geometric parameters (Å, º)

**Table:**

O1—C1	1.327 (3)	C4—H4A	0.9800
O1—C6	1.452 (4)	C4—H4B	0.9800
O2—C1	1.192 (3)	C4—H4C	0.9800
N1—C2	1.483 (3)	C5—H5A	0.9800
N1—H11	0.91 (3)	C5—H5B	0.9800
N1—H12	0.81 (4)	C5—H5C	0.9800
N1—H13	0.94 (3)	C6—C7	1.476 (5)
C1—C2	1.507 (4)	C6—H6A	0.9900
C2—C3	1.543 (4)	C6—H6B	0.9900
C2—H2	1.0000	C7—H7A	0.9800
C3—C4	1.516 (5)	C7—H7B	0.9800
C3—C5	1.531 (5)	C7—H7C	0.9800
C3—H3	1.0000		
			
C1—O1—C6	118.7 (3)	C3—C4—H4B	109.5
C2—N1—H11	113.3 (19)	H4A—C4—H4B	109.5
C2—N1—H12	112 (2)	C3—C4—H4C	109.5
H11—N1—H12	105 (3)	H4A—C4—H4C	109.5
C2—N1—H13	110.6 (18)	H4B—C4—H4C	109.5
H11—N1—H13	107 (3)	C3—C5—H5A	109.5
H12—N1—H13	108 (3)	C3—C5—H5B	109.5
O2—C1—O1	124.8 (3)	H5A—C5—H5B	109.5
O2—C1—C2	124.6 (2)	C3—C5—H5C	109.5
O1—C1—C2	110.6 (2)	H5A—C5—H5C	109.5
N1—C2—C1	108.1 (2)	H5B—C5—H5C	109.5
N1—C2—C3	110.0 (2)	O1—C6—C7	108.0 (3)
C1—C2—C3	113.6 (2)	O1—C6—H6A	110.1
N1—C2—H2	108.3	C7—C6—H6A	110.1
C1—C2—H2	108.3	O1—C6—H6B	110.1
C3—C2—H2	108.3	C7—C6—H6B	110.1
C4—C3—C5	111.0 (3)	H6A—C6—H6B	108.4
C4—C3—C2	113.4 (2)	C6—C7—H7A	109.5
C5—C3—C2	110.6 (3)	C6—C7—H7B	109.5
C4—C3—H3	107.2	H7A—C7—H7B	109.5
C5—C3—H3	107.2	C6—C7—H7C	109.5
C2—C3—H3	107.2	H7A—C7—H7C	109.5
C3—C4—H4A	109.5	H7B—C7—H7C	109.5
			
C6—O1—C1—O2	−4.0 (4)	N1—C2—C3—C4	57.1 (4)
C6—O1—C1—C2	173.9 (2)	C1—C2—C3—C4	−64.3 (4)
O2—C1—C2—N1	−33.1 (3)	N1—C2—C3—C5	−177.5 (3)
O1—C1—C2—N1	148.9 (2)	C1—C2—C3—C5	61.2 (4)
O2—C1—C2—C3	89.3 (3)	C1—O1—C6—C7	−170.3 (3)
O1—C1—C2—C3	−88.7 (3)		

### (2S)-1-Ethoxy-3-methyl-1-oxobutan-2-aminium chloride Hydrogen-bond geometry (Å, º)

**Table:**

*D*—H···*A*	*D*—H	H···*A*	*D*···*A*	*D*—H···*A*
N1—H11···Cl1^i^	0.91 (3)	2.34 (4)	3.192 (3)	157 (3)
N1—H12···Cl1^ii^	0.81 (4)	2.38 (4)	3.133 (3)	155 (3)
N1—H13···Cl1^iii^	0.94 (3)	2.19 (3)	3.122 (2)	175 (3)
C2—H2···O2^iv^	1.00	2.40	3.377 (3)	165
C7—H7*B*···Cl1^v^	0.98	2.82	3.760 (4)	160

Symmetry codes: (i) −*x*+1, *y*+1/2, −*z*; (ii) −*x*+1, *y*−1/2, −*z*; (iii) *x*−1, *y*, *z*; (iv) *x*, *y*−1, *z*; (v) −*x*+1, *y*+1/2, −*z*+1.
